# Pancreas Transplantation in Black, Asian and Minority Ethnic Patients-Single Centre Experience in the UK

**DOI:** 10.3389/ti.2022.10490

**Published:** 2022-06-15

**Authors:** Jeevan Prakash Gopal, Adam McLean, Jeremy Crane, Paul Herbert, Vassilios Papalois, Frank J. M. F. Dor, Anand Rathnasamy Muthusamy

**Affiliations:** ^1^ Imperial College Renal and Transplant Centre, Hammersmith Hospital, Imperial College Healthcare NHS Trust, London, United Kingdom; ^2^ Department of Surgery and Cancer, Faculty of Medicine, Imperial College London, London, United Kingdom

**Keywords:** pancreas transplantation, race, ethnicity, metabolic outcomes, SPK transplantation, Caucasian, BAME

## Abstract

Ethnic disparities in the outcomes after simultaneous pancreas kidney (SPK) transplantation still exist. The influence of ethnicity on the outcomes of pancreas transplantation in the UK has not been reported and hence we aimed to investigate our cohort. A retrospective analysis of all pancreas transplant recipients (*n* = 171; Caucasians = 118/Black Asian Ethnic Minorities, BAME = 53) from 2006 to 2020 was done. The median follow-up was 80 months. Patient & pancreas graft survival, rejection rate, steroid free maintenance rate, HbA1c, weight gain, and the incidence of secondary diabetic complications post-transplant were compared between the groups. *p* < 0.003 was considered significant (corrected for multiple hypothesis testing). Immunosuppression consisted of alemtuzumab induction and steroid free maintenance with tacrolimus and mycophenolate mofetil. Pancreas graft & patient survival were equivalent in both the groups. BAME recipients had a higher prevalence of type-2 diabetes mellitus pre-transplant (BAME = 30.19% vs. Caucasians = 0.85%, *p* < 0.0001), and waited for a similar time to transplantation once waitlisted, although pre-emptive SPK transplantation rate was higher for Caucasian recipients (Caucasians = 78.5% vs. BAME = 0.85%, *p* < 0.0001). Despite equivalent rejections & steroid usage, BAME recipients gained more weight (BAME = 7.7% vs. Caucasians = 1.8%, *p* = 0.001), but had similar HbA1c (functioning grafts) at 3-,12-, 36-, and 60-months post-transplant.

## Graphical Abstract

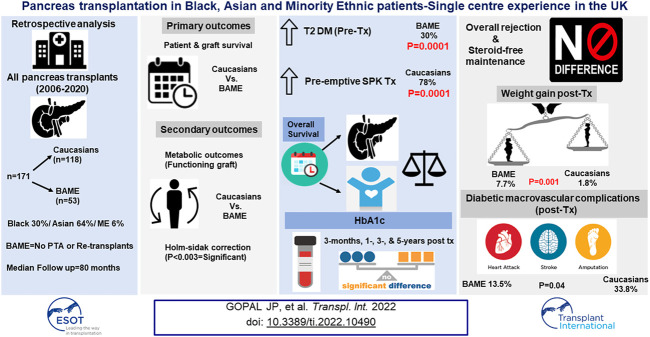

## Introduction

Despite increasing interest in equitable healthcare, disparities in access to solid organ transplantation, especially for ethnic minority patients, still exists (1, 2). Most of the literature on ethnicity-based outcomes in pancreas transplantation are from the USA (3–6), and the healthcare delivery in the USA is predominantly through insurance companies. There is no equivalent data from the UK, where the healthcare system is publicly funded. In view of this and in addition, as our centre serves an ethnically diverse patient population (7) (which corresponds to the geographical location and the ethnic spread in the locality), we aimed to investigate the influence of ethnicity on the outcomes of pancreas transplantation in our patient cohort, with a special focus on metabolic outcomes. This represents the first single center experience on ethnicity-based outcomes of pancreas transplantation from the UK.

## Materials and Methods

Following institutional audit committee approval, a retrospective analysis of all pancreas transplants (including simultaneous pancreas kidney-SPK, solitary pancreas, and re-transplants) performed between January 2006 and March 2020 was done. Data was collected from a prospectively maintained local database and National Health Service Blood and Transplant’s centre database.

### Donor Selection Criteria

According to our center’s organ acceptance policy, all the DBD donors were less than 65 years old and all the DCD donors were less than 55 years old. The donor’s body mass index (BMI) cut off was ≤30 kg/m^2^. All the DCD donors had a functional warm ischemia time (systolic blood pressure <50 mmHg and/or oxygen saturation of 70%) of less than 60 min and a downtime of less than 30 min.

### Immunosuppression

Immunosuppression consisted of induction with intravenous alemtuzumab 30 mg (single dose) and methylprednisolone 500 mg. Maintenance immunosuppression was with tacrolimus, mycophenolate mofetil, and a short course of steroids (7 days). Post-transplant target tacrolimus trough levels were 8–12 ng/dl.

### Criteria for Transplanting Type 2 Diabetes Mellitus

Patients were classified as type 2 diabetes mellitus if they have a detectable C-peptide and the classification was predominantly based on the diagnosis made by the referring diabetologist. The following is the criteria for transplanting type 2 diabetic patients: Insulin treated diabetes along with end stage renal failure with a body mass index of ≤30 kg/m^2^, glycaemic lability, and insulin requirement of less than 1 Unit/Kg/day.

### Outcome Measures Studied

All primary and secondary outcomes were compared between Caucasian and BAME recipients; the primary outcome measures were patient and pancreas graft survival, secondary outcome measures were metabolic outcomes among those with a functioning graft (weight gain, HbA1c, and incidence of secondary diabetic macrovascular complications post-transplant), rejection rate, and steroid usage between the two groups.

### Definition of Outcome Parameters

A functioning graft is defined as remaining insulin independent post-transplantation. Secondary diabetic macrovascular complications are defined as any of the following events post-transplant: myocardial infarction, cerebrovascular accident, transient ischemic attack, and/or limb amputations (minor or major). Rejection episodes are either cellular or antibody mediated or mixed, and comprise of either pancreas or kidney rejection (in case of simultaneous pancreas-kidney transplantation). The rejections defined are either biopsy proven or those episodes that were treated based on clinical suspicion (raising serum amylase, positive circulating donor specific antibody, or delayed pancreatitis on CT scan).

### Statistical Analysis

Categorical variables are expressed as frequency (%) and continuous variables as median. Differences between the categorical variables were assessed using Fisher’s exact test and differences between the continuous variables were assessed by using Mann Whitney test. Survival analysis was done by using Kaplan-Meir survival plots. Holm-Sidak correction was done for multiple comparisons and a *p* value of <0.003 was considered significant. All the statistical analyses were performed using Graph Pad Prism software (Version 9.0).

## Results

**FIGURE 1:**
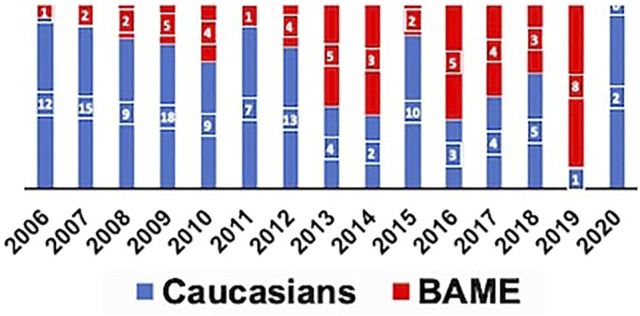
Proportion of Caucasian and BAME recipients transplanted each year.

A total of 171 pancreas transplants (SPK-129/PAK-27/PTA-4/Re-transplants-11) were performed during the study period of which 118 recipients were Caucasians and 53 were from the BAME group. Among the BAME group 64% (*n* = 34) were from Asian communities, 30% (*n* = 16) from Black communities, and 6% (*n* = 3) from Minority Ethnicities. The median follow-up period of the study was 80 months. The year-wise distribution of pancreas transplant activity in our center is shown in Figure 1. Donor and recipient characteristics is shown in Table 1. The HLA mismatch was grouped into 4 levels as per the NHSBT data description. The definition of each level is shown in Table 1.

**TABLE 1:** Donor and recipient characteristics.

Donor and recipient characteristics	Caucasians	BAME	*p* value
Donor age in years (Median)	35	32	0.39
Donor BMI in kg/sq. m (Median)	23.10	24.10	0.12
DCD donors (%)	12.71	16.98	0.48
Recipient age in years (Median)	45	41	0.02
Sensitized recipients % (CRF>5%)	20.34	24.53	0.55
Re-transplants	11	0	—
HbA1c at registration in mmol/mol (Median)	71.8	63.9	0.03
Duration of diabetes in years (Median)	30.50	23	<0.0001
Age at onset of diabetes in years (Median)	13	20	0.01
Pre-transplant insulin use (IU/Day)-Median	44	41.50	0.54
Solitary pancreas transplants-% (PAK/PTA)	27.12	13.21	0.05
Pre-transplant type 2 diabetes (%)	0.85	30.19	0.0001
Pre-transplant secondary diabetic macrovascular complications (%)	12.7	3.7	0.07
Pre-transplant registered blind (%)	10.17	13.21	0.55
eGFR at referral in ml/min (Median)	20	14.5	0.47
Waiting time in days (Median)	232	217	0.96
Time taken for workup in days (Median)	166	122	0.60
Cold ischemia time in mins (Median)	938	799	0.001
Pre-emptive SPK transplantation (%)	78	43	0.0001
HLA group 1 (0) & 2 (0DR+0/1B)	6%	—	0.06
HLA group 3 (0DR+2B) or (1DR+0/1B)	28%	32%	0.55
HLA group 4 (1DR+2B) or (2DR)	66%	68%	0.83

There was no pancreas transplant alone or re-transplants in the BAME cohort. The waiting time defined as the time from activation in the national transplant waiting list to transplantation were similar for both the groups, although the pre-emptive transplantation rate (for SPK transplantation) was significantly higher for Caucasian recipients. Data on workup time, which is the time from referral to our centre to activation in the national transplant waiting list, were available for 45 patients and were similar for both the groups. Data on estimated glomerular filtration rate at the time of referral for SPK transplantation were available for 51 patients and were similar for both the groups. There was a significantly higher prevalence of type 2 diabetes mellitus in the BAME group.

### Patient and Pancreas Graft Survival

The 1-, 3-, and 5-year patient survival were comparable between the two groups (Table 2). There were 22 early graft losses (within 90 days post-transplant) in total. The following were the causes for early graft loss; thrombosis (*n* = 6), bleeding (*n* = 6), sudden cardiac death (*n* = 3), pancreas failed to perfuse on table (*n* = 2), severe graft pancreatitis (*n* = 2), duodenal anastomotic leak (*n* = 1), Y-graft pseudoaneurysm (*n* = 1), and unknown (*n* = 1). The early graft losses were not significantly different between the two groups (Caucasians = 16.10%, *n* = 19 vs. BAME = 5.6%, *n* = 3, *p* = 0.05). The 1-, 3-, and 5-year pancreas graft survival for both SPK and isolated pancreas transplants (PAK/PTA) were comparable between the two groups (Table 2).

**TABLE 2:** Pancreas graft and patient survival by ethnicity.

Survival	Caucasians,%	BAME,%	Log rank p
1-year (SPK)-Pancreas	84.07	88.60	0.47
3-year (SPK)-Pancreas	77.02	85.84	0.36
5-year (SPK)-Pancreas	75.18	85.84	0.29
1-year (PAK)-Pancreas	54.54	100	0.04
3-year (PAK)-Pancreas	41.06	100	0.02
5-year (PAK)-Pancreas	41.06	75	0.03
1-year (Patient)	98.21	96.18	0.41
3-year (Patient)	93.72	84.42	0.08
5-year (Patient)	86.23	80.20	0.25

### Steroid-Free Maintenance and Rejection Rate

The overall rejection rates and steroid-free maintenance rates were comparable between the two groups (Caucasians = 18.1% vs. BAME = 22.6%, *p* = 0.49; Caucasians = 81.8% vs. BAME = 81.1%, *p* = 0.92, respectively).

### Metabolic Outcomes

**FIGURE 2:**
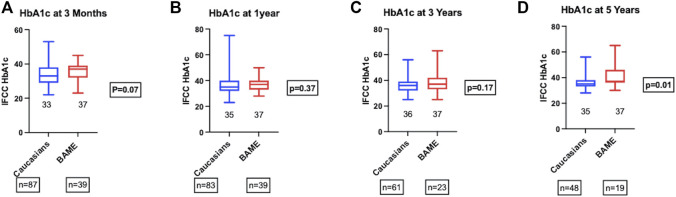
HbA1c at **(A)** 3-month, **(B)** 1-year, **(C)** 3-year, and **(D)** 5-year post-transplant.

The metabolic outcomes were compared in those recipients with a functioning graft. There were no significant differences in the HbA1c at 3-month, 1-, 3-, and 5-year post-transplant between the two groups (Figure 2). BAME recipients gained significantly more weight post-transplant compared to their Caucasian counterparts (Median percentage weight gain; BAME = 7.7% vs. Caucasians = 1.8%, *p* = 0.001). The overall incidence of secondary diabetic macrovascular complications post-transplant was not significantly different between the two groups (Caucasians = 33.8% vs. BAME = 13.5%, *p* = 0.04). There were 10 cardiovascular events (Caucasians = 8 vs. BAME = 2), 9 cerebrovascular events (Caucasians = 8 vs. BAME = 1), and 9 peripheral vascular events (Caucasians = 7 vs. BAME = 2).

### Type 1 vs. Type 2 Diabetes-Outcomes

A subgroup analysis was performed to look into whether type 2 diabetes mellitus had an impact on the ethnicity-based outcomes. The overall pancreas graft and patient survival were similar for pre-transplant type-1 and tye-2 diabetic patients (Log rank *p* = 0.04, and 0.98, respectively). The post-transplant HbA1c among those with a functioning graft were similar for both type-1 and type-2 diabetic patients at 3-month, 1-, 3-, and 5-year (*p* = 0.02, 0.01, 0.02, and 0.02, respectively). There was no significant difference in the post-transplant median percentage weight gain either (Type-1 diabetes = 3.1% vs. Type-2 diabetes = 7.7%, *p* = 0.07).

## Discussion

A successful pancreas transplantation leads to improvement in quality of life as well as improvement in cardiovascular risk profile, and reduction in macrovascular disease along with survival benefits in diabetic patients (8–12). Hence, the real argument for pancreas transplantation is to achieve optimal metabolic control whereas improving survival should be an added advantage. The studies reporting ethnicity-based outcomes in pancreas transplantation so far have not looked into the metabolic outcomes. As the prevalence of type-1 diabetes is less common in the non-Caucasian population (13), it is vital that metabolic outcomes should be considered alongside survival outcomes in this cohort. A review of data from the United Network for Organ Sharing (UNOS) database/Organ Procurement and Transplantation Network (OPTN) have reported that African-Americans have worse long-term survival rates (both patient and graft) compared to the other ethnic groups (3, 4). Access to pancreas transplantation has also been reported to be limited for African-Americans (5, 6), which could be due to a referral bias because of the presumed inferior outcomes in this patient group. In an era of increasing global immigration, it is crucial to avoid ethnic disparities in access to transplantation.

We noted equivalent patient and graft survival (for both SPK and solitary pancreas transplants) in Caucasian and BAME recipients. This is in contradiction to some of the major studies that have been published before (3, 4). From USA data, it transpires that Asians and Hispanics are reported to have the best survival outcomes and African-Americans have better short-term outcomes compared to Caucasians but poor longer-term outcomes (3). As Asian recipients comprised the majority of the BAME cohort in our centre, our results could be that of an Asian subtype rather than BAME community as a whole. It is also important to note that as the BMI cut off for Type 2 diabetic patients was 30 kg/sq.m, some of them might have non-classical type of diabetes rather than presumed type 2 diabetes mellitus (14). In the UK, minority groups are collectively referred to as BAME. Despite the fact that this terminology has been criticised due to the heterogeneity within the group, it is still widely in use. This heterogeneity might also explain similar survival outcomes observed in our study. Although this has been the case, our study supports the more recent observation based on data from the USA that outcomes are not necessarily inferior for non-Caucasian recipients (15–17).

HbA1c among those with a functioning graft was not significantly different between the two groups until 5 years post-transplant, although there is a trend towards higher HbA1c in BAME recipients at 5 years. Longer term follow-up data might uncover the effect of weight gain on HbA1c and graft survival. As HbA1c is known to be an independent predictor of long term pancreas graft failure (18), other metabolic parameters such as mixed meal tolerance test and C-peptide measurements were performed only selectively in patients with allograft dysfunction with consideration of intervention aimed at optimizing graft function and were not part of the routine follow up protocol.

Despite similar rejection rates and steroid usage in both the groups, BAME recipients gained significantly more weight post-transplantation. In our study we were unable to characterise whether the weight gain was due to an increase in lean body mass or due to increased adiposity. Peripheral hyperinsulinism resulting from systemic venous drainage has been postulated as a cause for excessive weight gain post-SPK transplantation (19). The explanation for higher weight gain in BAME recipients could only be speculative due to the retrospective nature of the study. Younger recipient age is known to be associated with weight gain post kidney and pancreas-kidney transplantation (20, 21). Weight gain post-transplantation has been observed in patients with a positive C-Peptide at the time of pancreas transplantation (22). The following could be the reasons for excessive weight gain in BAME group; BAME recipients were relatively younger compared to their Caucasian counterparts, and the majority of type-2 diabetics were from the BAME group. Longitudinal data on weight gain would be useful in identifying the time frame where recipients start to gain excess weight. This might help in planning dietary/behavioural modification or metabolic interventions such as the introduction of GLP-1 analogues in those at risk (23, 24). Additionally, the use of calcineurin inhibitors is known to cause insulin resistance thereby leading on to excessive weight gain (25, 26). Furthermore, it is well known that African-Americans need aggressive tacrolimus dosing to achieve optimal trough levels due to the ethnic difference in the prevalence of CYP3A5*3 genotype, which is responsible for the metabolism of tacrolimus (27, 28). Further studies looking at the circulating tacrolimus trough levels and metabolic parameters will provide more insight into strategies for optimal maintenance immunosuppression.

The incidence of pre-, and post-transplant secondary diabetic macrovascular complications were numerically higher in Caucasian recipients, although, statistically insignificant. There are several reasons for this observation. Firstly, Caucasians had an early onset and a significantly longer duration of diabetes compared to the BAME group. Hence Caucasian recipients had more macrovascular complications due to poor metabolic control. BAME recipients might have had a good metabolic control for a longer period than Caucasians before worsening control and hence the lower incidence of pre-transplant macrovascular complications. Secondly, this is also be due to a more conservative approach in listing Type 2 diabetic patients for pancreas transplantation, as is the case in BAME patients in whom Type 2 diabetes mellitus was more prevalent (29–31). Excessive weight gain observed in the BAME group could potentially lead on to post transplant metabolic syndrome and may increase the risk of cardiovascular complications in the longer term.

Prior to 2012, a majority of the patients transplanted were Caucasians and the cold ischemia times were longer. Post 2012, more BAME patients were being transplanted and the cold ischemia time was progressively shorter. Our centre’s change in practice reflected the evolving evidence base as convincing literature evidence was generated around the same time supporting pancreas transplantation in Type 2 diabetes mellitus (32–34). Due to the timeline effect there was a significant difference in the cold ischemia time between the two cohorts and there are no other explanations.

The pre-emptive transplantation rate was higher for Caucasian recipients, although, the estimated glomerular filtration rate (eGFR) at the time of referral, time taken for transplant work-up (time from referral to listing), and the waiting times were similar for both the groups. There can be several reasons for this. Most of the studies from around the world have reported that lower socio-economic status is independently associated with reduced access to pre-emptive listing (35–37). Despite a publicly funded healthcare system in the United Kingdom, socio-economic and geographic disparities in the utilisation of live donor kidney transplantation has been reported (38). Due to lack of data on socio-economic status; we are unable to comment further on that. The other reason could be the location of the patient and the proximity to a transplant centre. Being registered with a transplanting centre is known to be associated with higher pre-emptive listing because of the onsite availability of specialist services for assessing suitability. Health literacy is another important factor. Involvement of BAME ambassadors in the discussion about transplantation might reduce the socio-cultural, and language barrier and also may improve the engagement rate of BAME patients to transplantation services. The use of social media and interactive ways of reaching out, rather than traditional written pamphlets about organ donation and transplantation might also improve the awareness among BAME patients. Further multicentre studies will shed more light on BAME access to pancreas transplantation and outcomes, in addition to centre variation in practice in the UK.

This is the first study from the UK reporting ethnicity-based outcomes in pancreas transplantation and the first study reporting metabolic outcomes in Caucasian and BAME patients. Type-2 diabetes was more prevalent in BAME patients. BAME and Caucasian recipients had similar HbA1c until 5 years post-transplantation. Despite similar rejection rates and steroid usage, BAME recipients gained more weight post-transplantation. BAME patients experience similar survival outcomes (graft and patient) to those of Caucasian recipients. Although the waiting time and work-up time were similar, Caucasians had a higher proportion of pre-emptive SPK transplantation.

## Data Availability

The original contributions in the study are included in the article/Supplementary Materials, further inquiries can be directed to the corresponding author.
